# Structural Features of the Nucleosomal DNA Modulate the Functional Binding of a Transcription Factor and Productive Transcription

**DOI:** 10.3389/fgene.2022.870700

**Published:** 2022-05-13

**Authors:** Vinesh Vinayachandran, Purnima Bhargava

**Affiliations:** Centre for Cellular and Molecular Biology (Council of Scientific and Industrial Research), Hyderabad, India

**Keywords:** chromatin structure, Nhp6, pol III, rotational phase, T_7_ element, U6 snRNA, transcription

## Abstract

A small non-histone protein of budding yeast, Nhp6 has been reported to specifically influence the transcription of a yeast gene, *SNR6*. The gene is essential, transcribed by the enzyme RNA polymerase III, and codes for the U6snRNA required for mRNA splicing. A translationally positioned nucleosome on the gene body enables the assembly factor TFIIIC binding by juxtaposing its otherwise widely separated binding sites, boxes A and B. We found histone depletion results in the loss of U6 snRNA production. Changing the rotational phase of the boxes and the linear distance between them with deletions in 5 bp steps displayed a helical periodicity in transcription, which gradually reduced with incremental deletions up to 40 bp but increased on further deletions enclosing the pseudoA boxes. Nhp6 influences the transcription in a dose-dependent manner, which is modulated by its previously reported co-operator, an upstream stretch of seven T residues centered between the TATA box and transcription start site. Nhp6 occupancy on the gene *in vivo* goes up at least 2-fold under the repression conditions. Nhp6 absence, T_7_ disruption, or shorter A–B box distance all cause the downstream initiation of transcription. The right +1 site is selected with the correct placement of TFIIIC before the transcription initiation factor TFIIIB. Thus, the T_7_ sequence and Nhp6 help the assembly and placement of the transcription complex at the right position. Apart from the chromatin remodelers, the relative rotational orientation of the promoter elements in nucleosomal DNA, and Nhp6 regulate the transcription of the *SNR6* gene with precision.

## Introduction

The packaging of the eukaryotic genome into chromatin affects all the DNA-templated processes. The *in vivo* chromatin structure often reflects on the recent transcription activity of a locus. Nucleosomal arrays are non-randomly punctuated by the nucleosome-free regions (NFRs), which are generally hotspots of high transcription activity, promoter and enhancer elements, replication origins, fragile genomic sites, etc. The U6 snRNA gene is one of the few examples where positioned nucleosomes have been shown to cause its transcriptional activation (Stnkel et al., 1997; Bhargava 2013). The gene is transcribed by RNA polymerase (pol) III, which transcribes short, non-coding genes such as 5S rRNA, U6snRNA (Didychuk et al., 2018), and tRNAs, which form the bulk of the pol III transcriptome. Although yeast tRNA genes are found in the NFR (Kumar and Bhargava 2013), the chromatin structure around these genes is shown to have a regulatory influence on their transcription (Shukla and Bhargava 2018). The genes characteristically have intragenic promoter elements, boxes A and B (typically 50–60 bp apart in tRNA genes), to which the transcription factor (TF) IIIC binds in the first step and recruits the initiation factor TFIIIB in the next step, and pol III joins next (Geiduschek and Kassavetis 2001). Correct positioning of TFIIIB, for which box A is important, decides the transcription start site (TSS) to be selected (Gerlach et al., 1995).

**FIGURE 1:**
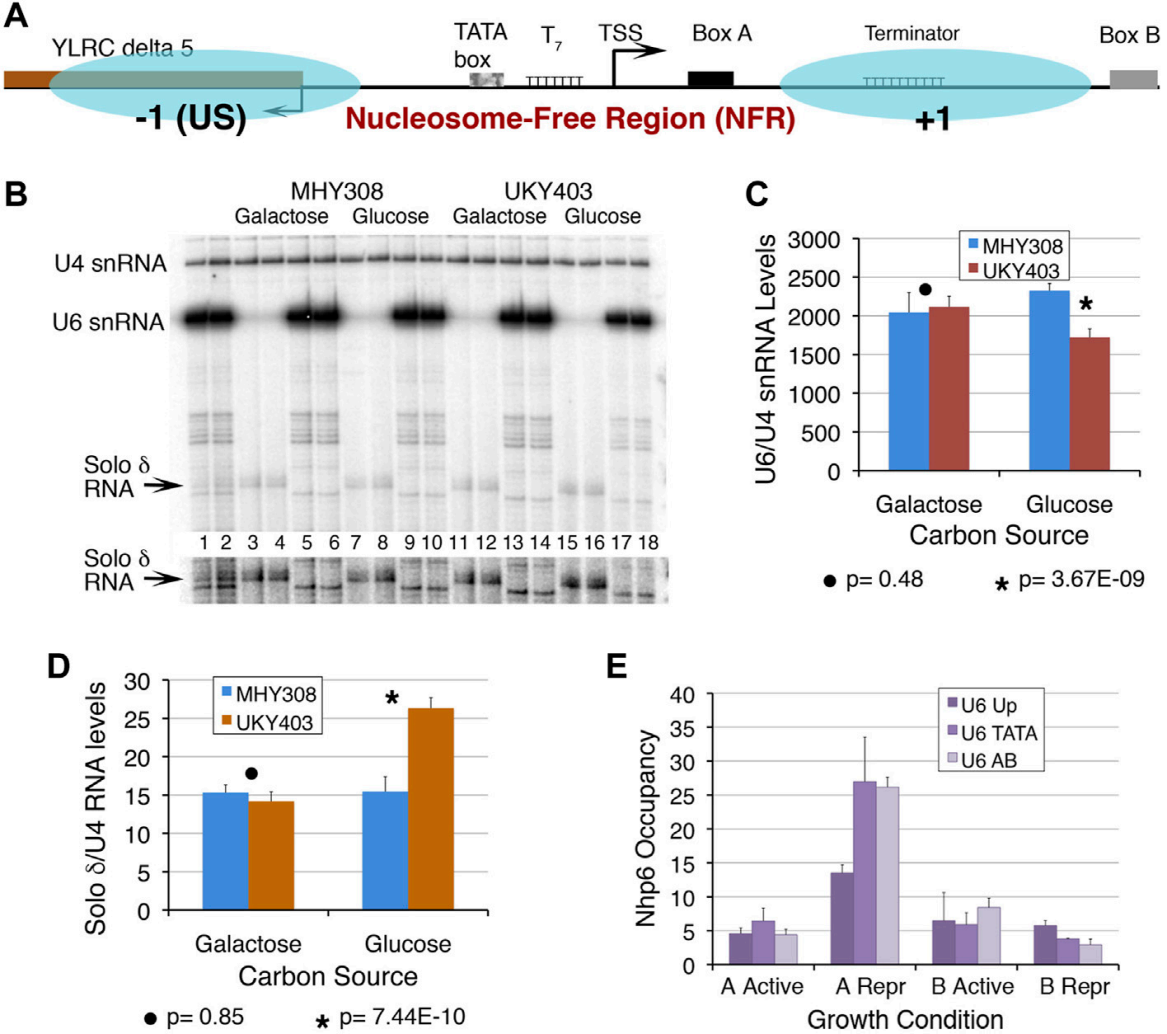
Chromatin at the *SNR6* locus affects the U6 snRNA levels. **(A)** Schematic representation of the *SNR6* locus. Blue ovals show two positioned nucleosomes mapped earlier in the gene region (Arimbasseri and Bhargava 2008). Box B is found at the 3′-end of the gene body (+1) nucleosome whereas the −1 nucleosome is found upstream (US) of the TATA box at the −30 bp position. The TATA box, T_7_ element, TSS (bent arrow), and box A are found in the NFR region. The US nucleosome by virtue of blocking the 5′ end of the Ty1 solo *δ* element (*YLRCδ5*) represses its expression. **(B)** A typical gel showing the primer extension products from duplicate samples. Disruption of the chromatin structure perturbs transcription at the *SNR6* locus. Yeast strains MHY308 and UKY403 (Supplementary Table S1) carry histone H4 genes under their own promoter or the *GAL1* promoter. When UKY403 cells are shifted to glucose for growth, H4 is depleted causing a loss of 50–60% of nucleosomes (Kim et al., 1988) by the time the cells get arrested. Cells were grown and processed for total RNA extraction as described earlier (Arimbasseri and Bhargava 2008). RNA was measured by the primer extension method using end-labeled gene-specific primers for cDNA synthesis in three independent experiments. U4 snRNA (pol II-transcribed) levels were used as the normalizer. Lanes 1 and 2 show primer extension products on total RNA from MHY308 and UKY403 using primers for U4, U6 snRNAs, and solo *δ* RNA, added together in the same extension reaction. Alternate pairs of the remaining lanes received primers of either solo *δ* (lanes 3, 4, 7, 8, 11, 12, 15 and 16) or U6 (lanes 5, 6, 9, 10, 13, 14, 17 and 18) along with the probe for U4 snRNA, which was used as the normalizer. A higher exposure of the gel area, cropped to visualize the solo *δ* RNA better, is given below the gel image. The significance of changes was confirmed by Student’s two-tail *t*-test. The *p* values are given below the graphs. Quantification results for U6 snRNA **(C)** and solo *δ* RNA **(D)** showing average levels and scatter for four biological replicates. A large difference in the *y* axis scale is due to the very low level of solo *δ* RNA. **(E)** Occupancies of Nhp6A and B were measured at three parts of the *SNR6* locus in cells expressing HA-tagged Nhp6 A or B proteins. ChIP sample preparation, real-time PCR primers, amplicons, and fold enrichment calculation using TELVIR as the normalizer were as described earlier (Arimbasseri and Bhargava 2008). Samples were prepared from cells grown in an enriched medium (active) or under nutrient-deprivation (Repr; repressed) conditions.

The yeast U6snRNA (*SNR6*) gene has an unusual organization (Figure 1A) in having an upstream TATA box and an unusually long linear distance (202 bp) between box A and extragenic box B found downstream of the gene terminator (Brow and Guthrie 1990; Eschenlauer et al., 1993). The TATA box enables the TFIIIC-independent recruitment of TBP-containing TFIIIB and naked DNA (ND) transcription on *SNR6*. However, TFIIIC binding to boxes A and B is absolutely essential for chromatin transcription (Burnol et al., 1993). A positioned nucleosome brings the two boxes closer in space, situating them near the entry and exit points of DNA in the nucleosome (Shivaswamy et al., 2004; Arimbasseri and Bhargava 2008). Additionally, a stretch of 7 T residues, the T_7_ element, centered between the TATA box and TSS (Figure 1A) is reported to support the role of a small non-histone protein Nhp6 in the pre-initiation complex (PIC) assembly on *SNR6* (Martin et al., 2001). Out of all pol III targets, yeast Nhp6 was shown to specifically influence *SNR6*. It activates the transcription of *SNR6* *in vitro* and *in vivo* (Kruppa et al., 2001; Lopez et al., 2001; Martin et al., 2001). On tRNA genes, Nhp6 was shown to improve the fidelity of transcription and loading of the basal transcription factor TFIIIC (Kassavetis and Steiner 2006) with a reduction of non-specific transcriptions. Nhp6 was also found to influence the transcription of a subset of tRNA genes in a dose-dependent manner (Braglia et al., 2007). However, none of the studies probed the role of Nhp6 in the chromatin context, and the mechanism by which Nhp6 specifically activates *SNR6* remains unclear.

Nhp6 was reported to promote the pol II PIC assembly *in vivo* (Paull et al., 1996). Both Nhp6 and positioned nucleosomes are reported to influence the pol III PIC assembly involving the correct placement of TFIIIB and TFIIIC on the U6 snRNA gene (Kruppa et al., 2001; Lopez et al., 2001; Martin et al., 2001; Zhao et al., 2001; Shivaswamy and Bhargava 2006). The relative spatial orientation and distance between A and B boxes may influence the stability of simultaneous TFIIIC binding to them. As opposed to earlier genetic and *in vitro* transcription experiments, in this study, the role of Nhp6 in the transcriptional activation of *SNR6* is investigated under the aforementioned two conditions in the chromatin context. The distance between A and B boxes was reduced in 5 bp increments, which generated a shorter distance and a helical phase difference between them, causing a gradual reduction of transcription. We found that Nhp6 activates TFIIIC-dependent chromatin transcription in a T_7_ stretch- and dose-dependent manner. Nhp6, together with the TATA box, T_7_ element, and optimal distance between A and B boxes rightly positions the TFIIIC and TFIIIB, which results in accurate TSS selection along with transcriptional activation.

## Materials and Methods

### Yeast Strains and Plasmid Templates

Yeast strains are described in Supplementary Tables S1. A total of 15 plasmids (named d5-d70 and dT_7_) were derived from the plasmid pCS6 (Supplementary Figures S1A, B and Supplementary Table S2). Three of them, d25, d35, and d70 were not used for most of the experiments because of very low transcription from them. The histone H4 depletion strain UKY 403 and control strain MHY308 (gifts from Michael Grunstein) were grown till 0.8 OD_600nm_ in YEPGal and then in YEPD for 3 h before harvesting and RNA extraction as described earlier (Arimbasseri and Bhargava 2008).

### ChIP and Real-Time Polymerase Chain Reaction

Yeast Nhp6A and B were HA-tagged at the C-terminal using the PCR toolbox (Janke et al., 2004). Both strains were used to measure Nhp6 occupancy over *SNR6* by using the ChIP and real-time PCR method (Arimbasseri and Bhargava 2008) as described earlier.

### DNA Templates and *in vitro* Transcription

The recombinant Nhp6A protein, with the N-terminal 6XHis-tag, was purified using an overexpression clone (gift from David Stillman, United States). The chromatin was assembled using the well-established *Drosophila* embryonic S-190 extract system, which gives equally spaced nucleosomal arrays over plasmids (Shivaswamy et al., 2004). The *in vitro* transcription using lab stocks of pure proteins TFIIIC, pol III, and recombinant TFIIIB was carried out as described in detail earlier and the transcripts were visualized by the primer-extension method (Shivaswamy et al., 2004). All transcript yields were normalized with corresponding levels from pCS6 in each experiment. At least three or more independent experiments were performed for all the measurements. The *p*-values were calculated by two-tailed Student’s t-test.

## Results

### Chromatin is an Integral Part of *SNR6* Transcription *in vivo*

A positioned nucleosome between boxes A and B of the *SNR6* gene was shown to enable the binding of TFIIIC and high transcriptional activation *in vitro* (Shivaswamy et al., 2004). The nucleosome positioned upstream (US) of the TATA box is regulatory in nature (Arimbasseri and Bhargava 2008), where it also blocks the 5′end of a solo *δ* element (*YLRCdelta5*) (Figure 1A). The PIC assembly occurs in the NFR, which encompasses the TATA box, TSS, T_7_ element, and box A (Figure 1A). We had earlier reported the loss of the overall chromatin organization at the *SNR6* locus under histone depletion conditions (Arimbasseri and Bhargava 2008). We found that under this condition, U6 snRNA levels are significantly reduced whereas the upstream solo *δ* element (pol II transcribed) is activated (Figures 1B–D), confirming that *SNR6* transcription requires a properly configured chromatin organization *in vivo*.

Nhp6, a protein belonging to the HMG1 class has two 89% identical isoforms in yeast Nhp6A and B (Stillman 2010). The Nhp6 presence has been reported earlier on the *SNR6* and some tRNA genes *in vivo* (Braglia et al., 2007). Our Nhp6 occupancy measurements by the ChIP and real-time PCR method found a similar enrichment of Nhp6A and Nhp6B on the TATA box and A–B box region of the *SNR6* gene locus (Figure 1E). Under starvation, the repression of pol III transcription (Moir and Willis 2013) is found to be accompanied by increased occupancy of specifically Nhp6A and a small loss of Nhp6B on the *SNR6* gene (Figure 1E). This suggests a repressive role of Nhp6A and a differential, non-redundant role of the Nhp6 isoforms on the *SNR6* gene.

### Distance Between Boxes A and B Affects Transcription of the *SNR6* Gene

**FIGURE 2:**
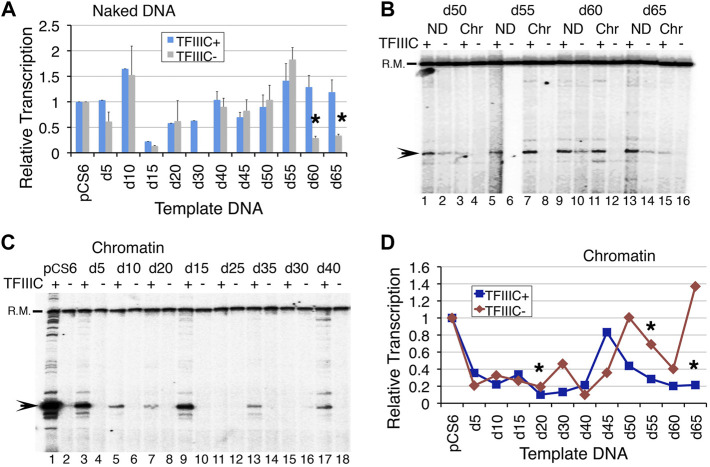
Reducing the distance between boxes A and B in 5 bp increments affects *SNR6* transcription. Plasmids as naked DNA or assembled into chromatin were used as templates for *in vitro* transcription assays with or without the addition of pure TFIIIC. A radiolabeled probe of a non-U6 sequence externally added before transcript extraction was used as the recovery marker (R.M.) and normalizer. Positions of R. M. and U6 transcript are marked on the left-hand side of the gel images. Gels were scanned in a PhosphorImager machine and the Image Guage (Fuji) software was used for quantifications of the transcripts. **(A)** Average and scatter of the measured U6 transcripts from three independent experiments. All levels were normalized against the respective pCS6 transcription levels in the absence/presence of TFIIIC. Asterisks mark the significant differences between the TFIIIC- and TFIIIC + transcription for d60 (*p* = 0.0016) and d65 (*p* = 0.0178). **(B)** Comparison of transcription from chromatin (Chr) and naked DNA (ND) in the presence/absence of TFIIIC. ND shows transcripts from all but the chromatin is not equally repressed in each case. **(C)** Comparison of the transcription from chromatinized deletion clones in the presence and absence of TFIIIC. **(D)** Comparison of quantifications of chromatin transcription shows different expressions from all the deletion clones. All measurements were normalized against the respective pCS6 levels. Asterisks mark the significant changes for d20 (*p* = 0.014), d55 (*p* = 0.023), and d65 (*p* = 0.004).

The deletion of 30–45 bp resulted in partial removal whereas longer deletions of 50 bp upwards in the complete removal of the pseudoA boxes (Supplementary Table S2). As expected, reducing the distance between the binding sites of TFIIIC resulted in somewhat periodic up and down levels of transcription (Supplementary Figure S1C), which reflect the changing helical phase of the DNA with deletions in 5 bp steps. TFIIIC- in dependent transcription was lowest for d15, d30, d60, and d65, while d10 and d55 were higher than pCS6 with/without TFIIIC (Figure 2A). The rest of the deletion clone NDs could be similarly transcribed with/without TFIIIC addition, staying below the pCS6 level (Figure 2A). This is not surprising since the transcription of *SNR6* ND is TFIIIC-independent. TFIIIC is known to slightly inhibit the naked pCS6 transcription. However, deleting one helical turn immediately next to the pseudoA boxes in the d10 plasmid gives ∼1.5-fold gain of transcription, whereas deletions of 5 bp or more than 15 bp up to 50 bp deletion, return only ∼60–90% of pCS6 transcription levels (Figure 2A). Interestingly, the transcription of d55–d65 increases to more than pCS6 levels with TFIIIC (Figure 2B, Supplementary Figure S1C, D). More than 50 bp deletions may reduce the linear distance between A and B boxes, but also constrain the steric flexibility of the intervening DNA, turning them out of phase on looping. Accordingly, d40–d55 are similarly transcribed with/without TFIIIC, and TFIIIC-dependent transcription increases for d55 whereas TFIIIC-independent transcription decreases for d60 and d65 with respect to the pCS6 level (Figures 2A,B, Supplementary Figures S1C, D). The increase on longer deletions with the deletion of the pseudoA boxes (Supplementary Figure S1A; Supplementary Table S2), suggests that the reduced TFIIIC-dependent transcription of pCS6 could be due to the sequestration of TFIIIC by the pseudoA boxes.

### Phasing out of Boxes A and B Affects Transcription of the *SNR6* Chromatin

The chromatin transcription of the deletion clones with and without TFIIIC showed an undulating pattern (Figures 2B–D, Supplementary Figures S1E, F) with a gradual decrease of TFIIIC-dependent transcriptional activation (Figure 2D, Supplementary Figure S1E). A decrease in transcription was seen followed by an increase with every 5 bp deletion in the next step up to 50 bp deletions. As a 5 bp deletion reduces the distance from optimal to less optimal, boxes A and B also fall out of phase with each other. With the next 5bp deletion, the boxes may again come in phase, resulting in a gain of transcription, although not to the original level. Therefore, an alternating decrease and increase suggests a change in the phase as the reason behind the pattern, which could directly influence the simultaneous binding of the multi-subunit TFIIIC to its two widely separated binding sites.

Earlier studies reported that a 42-bp deletion between the terminator and B box (Δ42) reduces transcription from *SNR6* more than an 84-bp deletion (Δ84) could (Eschenlauer et al., 1993). In agreement with this, transcription was found at very low levels when 20–40 bp were deleted (Figures 2C,D), with the lowest observed levels from the d40 plasmid (Supplementary Figure S1E). Moreover, although TFIIIC-independent transcription increased with further deletions, the TFIIIC-dependent chromatin transcription remained lower than the pCS6 level (Figures 2B,D, Supplementary Figure S1F). In the absence of TFIIIC, the highest transcription was seen from d50, on both the chromatin and ND, but the highest activation was on the d45 chromatin (Figure 2D, Supplementary Figure S1E). Surprisingly, transcription from the d20, d30, and d50–d65 chromatin remains repressed with TFIIIC addition, suggesting severe compromise of TFIIIC binding to these templates (Figures 2B–D, Supplementary Figure S1E). The aforementioned results show a very subtle effect of the intervening DNA in the transcription of *SNR6* according to the gap length, DNA phase, and hence, the orientation of TFIIIC binding sites as discussed later. The results agree with earlier studies suggesting TFIIIC-dependence of transcriptional activation by Nhp6.

### Nhp6 Increases Fidelity and TFIIIC-Dependent Transcription

**FIGURE 3:**
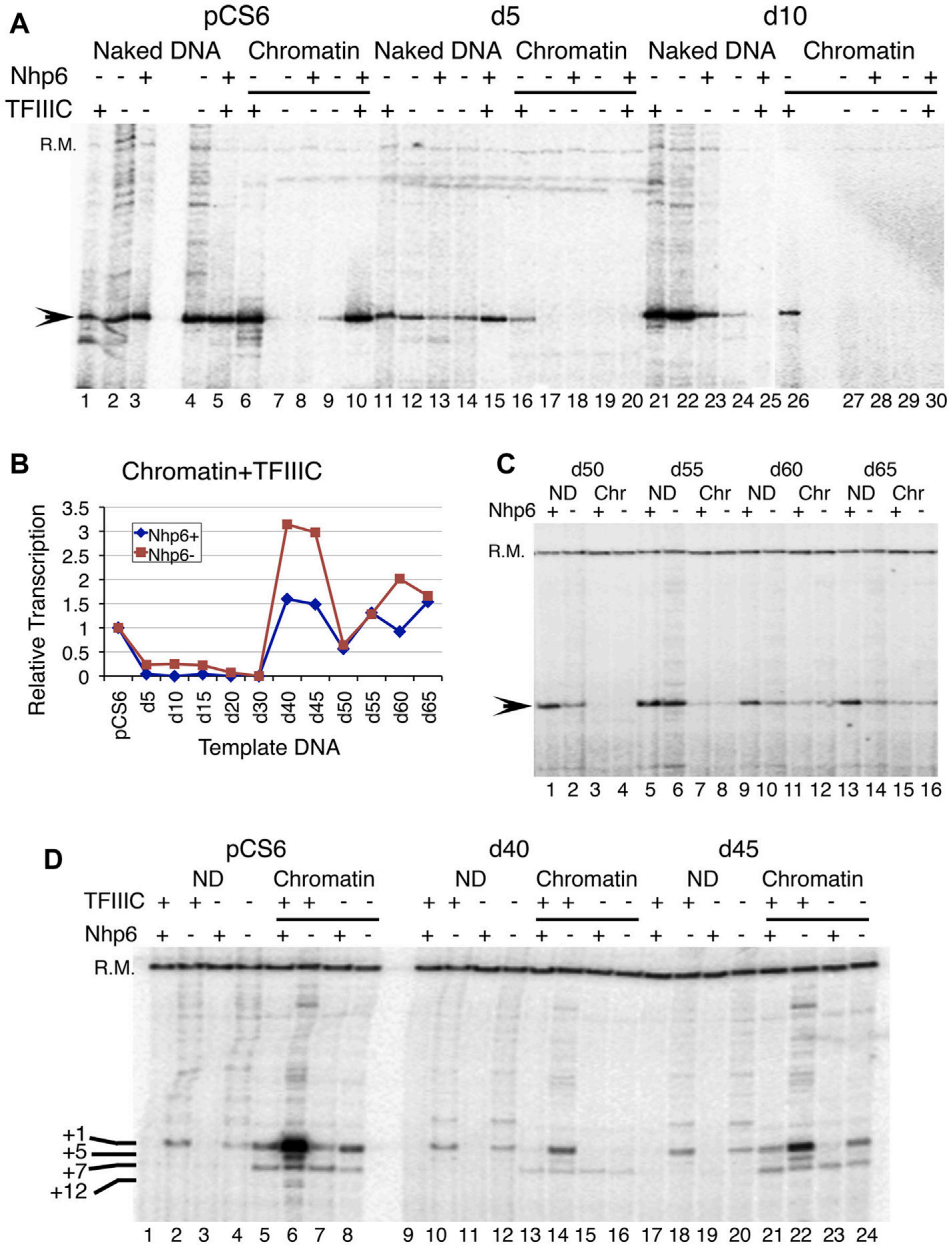
Effect of Nhp6 on transcription of deletion clones. **(A)** Effect of a 180-ng Nhp6 addition on the transcription of naked and chromatinized pCS6, d5, and d10 plasmids *in vitro*. Nhp6 supports the TFIIIC-dependent transcription of pCS6 but not of d5 and d10. **(B)** Quantification results of Nhp6 effect on the chromatin transcriptions of pCS6 and all deletion clones in the presence of TFIIIC with (180 ng) and without Nhp6 are plotted. Measured transcript levels were obtained by first normalizing with the recovery marker (R.M.) and then with the corresponding pCS6 levels. **(C)** TFIIIC-dependent transcriptions of d50-d65 ND and chromatin (Chr) are compared in the presence (108 ng) and absence of Nhp6. **(D)** Comparison of transcription from the ND and chromatin pCS6, d40, and d45 templates with/without TFIIIC are shown. Added amount of Nhp6 was 120 ng. The recovery marker (R.M.) and downstream-initiated transcripts from +5, +7, and +12 bp positions are marked along with the +1 transcript on the left-hand side of the panel.

Nhp6 showed a dose-dependent effect on the *in vitro* chromatin transcription of a tRNA gene (Mahapatra et al., 2011). We found that the addition of 60 ng Nhp6 activated two of the templates, d40 and d45, more than two-fold (Supplementary Figure S2A). As the Nhp6 amount is increased further, chromatin activation in the presence of TFIIIC is reduced (Supplementary Figure S2A), suggesting that Nhp6 influences *SNR6* transcription in a dose-dependent manner. Nhp6 is reported to work through the stabilization of the TFIIIC-DNA complex (Kassavetis and Steiner 2006), which is essential for chromatin transcription. Much of the non-specific transcription from naked pCS6 is suppressed in the presence of TFIIIC or Nhp6, which together increased the initiation from the +1 site (Figure 3A, lanes 1–5). Similar to previous reports (Lopez et al., 2001), Nhp6 gave a 1.5- to 2-fold increase of the naked pCS6 transcription but inhibited d5-d15 ND or chromatin transcription with/without TFIIIC (Figures 3A,B, Supplementary Figure S2B). ND transcription of *SNR6* with further deletions could not be enhanced by Nhp6 without/with TFIIIC addition (Figure 3C, Supplementary Figures S2C, D). Surprisingly, Nhp6 activated d50 and d65 ND transcription by ∼2- to 2.5-fold in the presence of TFIIIC (Figure 3C, Supplementary Figure S2C); their chromatin form is not activated by TFIIIC (Figure 2D).

### Nhp6 Reduces TFIIIC-dependent Activation of Chromatin Transcription

No activation of chromatin by Nhp6 could be seen in the absence of TFIIIC (Figures 3A–C). TFIIIC binding to the repressed *SNR6* chromatin results in its high activation (Shivaswamy et al., 2004). On pCS6, ∼10-fold TFIIIC-dependent activation of transcription is inhibited to ∼2.4-fold with Nhp6 addition (Figure 3D, lanes 5 and 6). As compared with the pCS6 chromatin, comparatively lower activation with TFIIIC (Figure 2) is further reduced on shorter deletion clones by Nhp6 (Figures 3A,B). While on longer deletion clones, Nhp6 addition to the d40, d45, and d60 chromatin reduced the TFIIIC-dependent activation to almost pCS6 level, and d30, d50, and d65 were unaffected (Figures 3B–D). One reason for the observed differences in the Nhp6 effect on the longer deletion clones (Figure 3B) could be the differential effects of Nhp6 on their ND transcription (Supplementary Figure S2C).

We also noticed that chromatin formation on the deletion plasmids suppressed the +1 transcription, giving a downstream initiated transcript instead, which is seen in all the conditions (Figure 3D, chromatin lanes). On the d50 plasmid, which showed the lowest (of all longer deletion clones) activation of chromatin transcription with TFIIIC (Figures 2B, 3C, Supplementary Figure S2E), Nhp6 addition could not restore the transcription from the right TSS (+1 transcript). The persistence of downstream initiation of transcription from the +7 bp position suggests altered TFIIIC and hence, TFIIIB placement upstream, which has been earlier suggested as the cause of different TSS selections in TATA box–A box double mutants (Eschenlauer et al., 1993).

The aforementioned results demonstrate that the Nhp6 effect is stronger on longer deletion clones where the pseudoA boxes are deleted and it generally represses the chromatin transcription in a TFIIIC-dependent manner. It appears that the pseudoA boxes may be serving as a guide to TFIIIC for binding to the upstream, right A boxes. Therefore, with a perturbation in TFIIIC binding in their absence, chromatin activation and right +1 site selection are both compromised on the plasmids d45–d65.

### Nhp6 Requires the T_7_ Promoter Element for Transcriptional Activation of *SNR6*

**FIGURE 4:**
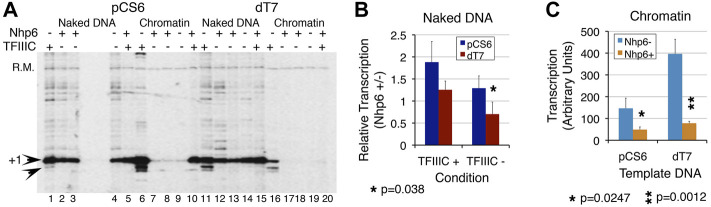
T7 promoter element is required for transcription repression effects of Nhp6. Transcription of pCS6 and dT_7_ was followed with or without Nhp6 addition (Nhp6+/−) in the presence/absence of TFIIIC. Measured transcript levels were obtained by normalizing with the recovery marker and the ratios of transcript levels in the presence/absence of Nhp6 were obtained separately for the transcription performed with/without TFIIIC (TFIIIC + or TFIIIC-) addition. **(A)** Nhp6 effect was followed with the addition of 108 ng Nhp6 in the lanes 3, 5, 8, and 10 for pCS6 and 13, 15, 18, and 20 for dT_7_ templates. Arrowheads mark the position of transcripts initiated at +1 and +5 bp positions. Nhp6 abolishes background, non-specific transcription seen only in the presence of TFIIIC. The results were analyzed for ND and chromatin separately. Read-out of the Nhp6 effect for naked DNA **(B)** is given as the ratio of transcript yield in the presence/absence of Nhp6 (Nhp6 +/-). Values of ratios less than 1 denote repression by Nhp6 whereas those more than 1 denote activation, over the transcription level in the absence of Nhp6. The *p* values for measurements from 3–4 independent experiments are given; *p* value 0.038 compares the transcript levels from pCS6 and dT_7_ in the absence of TFIIIC. In contrast to ∼1.29-fold (*p* = 0.13) activation for pCS6, dT_7_ transcription shows further 44% loss in the TFIIIC absence (*p* = 0.032). **(C)** Close to background chromatin transcription in the absence of TFIIIC gives high scatter in measurements. Because of this, only TFIIIC-dependent transcription in the absence or presence of Nhp6 was quantified. Nhp6 addition reduced the transcription similarly for pCS6 (3-fold, *p* = 0.0247) and dT_7_ (∼5-fold, *p* = 0.0012). Asterisk marks the significant differences; the *p* values are given at the bottom of the panels.

The T_7_ promoter element, positioned between the TATA box and TSS is reported to co-operate with Nhp6 in the transcriptional activation of yeast *SNR6* (Martin et al., 2001). The chromatin transcription shows higher sensitivity to Nhp6 levels (Figure 3). Nhp6 clearly showed stronger inhibition of pCS6 than dT_7_ transcription in a dose-dependent manner (Supplementary Figure S3), suggesting that the T_7_ sequence may not be required for normal transcription but enhances the effects of Nhp6 on *SNR6*. In the presence of TFIIIC, Nhp6 suppresses the downstream transcription initiation from the pCS6 chromatin and dT_7_ ND templates (Figure 4A, lanes 6 vs. 10 and 11 vs. 15). Consistent with the previously reported role of Nhp6 in increasing the transcriptional fidelity of PoI III on tRNA genes (Kassavetis and Steiner 2006; Mahapatra et al., 2011), Nhp6 could abolish downstream initiation of the pCS6 ND and chromatin. In contrast, transcription was completely inhibited by Nhp6 on the dT_7_ chromatin (Figure 4A), suggesting a role for T_7_ deletion in the chromatin repression. Thus, apart from the reported roles of TATA and A boxes (Gerlach et al., 1995), the T_7_ stretch promoter element may also have a role in the TSS selection and TFIIIC binding.

As compared with ∼1.9-fold Nhp6-dependent activation of ND transcription on pCS6, T_7_ disruption returned only ∼1.25-fold (*p* < 0.1) activation in the presence of TFIIIC (Figure 4B). With respect to pCS6+TFIIIC, ∼0.7-fold (*p* = 0.0082) activation for dT_7_ -TFIIIC resulted in repression (Figure 4B). TFIIIC absence and T_7_ disruption influence Nhp6 similarly. Additive effects of the three components demonstrate that both Nhp6 and the T_7_ element co-operate with TFIIIC to activate transcription on ND.

The Nhp6 effects on the TFIIIC-dependent dT_7_ chromatin and ND transcription activation were opposite. The T_7_ disruption gave ∼2.7-fold gain (*p* = 0.006) of transcription in the absence of Nhp6 (Figure 4C), whereas Nhp6 addition significantly reduced this gain (cf. pCS6 and dT_7_, Nhp6+ condition, Figure 4C) to only ∼1.6-fold (*p* = 0.028), indicating a reduced TFIIIC binding to the dT_7_ chromatin. Consistent with an earlier report (Martin et al., 2001), the results show that Nhp6 requires the T_7_ element to manifest its influence fully on the transcription of *SNR6*.

The aforementioned results show that the T_7_ sequence regulates the dose-dependent effects of Nhp6 on the TFIIIC-dependent chromatin transcription of the *SNR6* gene. Taken together, this study has demonstrated that reducing the distance by short 5–40 bp deletions between the terminator and box B does not improve transcription; a longer deletion including extragenic pseudoA boxes does. Chromatin transcription from yeast *SNR6* is activated at lower and repressed at higher Nhp6 levels. Nhp6 increases transcription fidelity by abolishing non-canonical initiations in favor of +1 transcription. This transcriptional activation depends on TFIIIC and the cis promoter element T_7_ stretch. Occupancy of specifically the Nhp6A isoform on the gene goes up under repression, attributing a repressive role to Nhp6 in keeping the highly active *SNR6* gene expression under check *in vivo*.

## Discussion

### Distance Between Boxes A and B Influences Transcription From Chromatin

Reducing the distance between the terminator and box B may constrain the TFIIIC binding whenever the A–B boxes do not fall in phase. For a particular DNA sequence wound over the nucleosome surface, rotational positioning decides the DNA phase accessible for a DNA-binding factor (Albert et al., 2007), while proximity of the two far apart binding sites may become possible by the looping out of intervening naked DNA (Bhargava and Chaterji, 1992) and winding of nucleosomal DNA (Pusarla et al., 2007). The *SNR6* gene sequence directs the assembly of nucleosomes with unique rotational settings on the whole gene (Vinayachandran et al., 2009). The nucleosome between boxes A and B, which is both rotationally and translationally placed on the gene body, gives a clear 145-bp nucleosomal footprint (Shivaswamy and Bhargava 2006). Considering the possibility of change in this position with reduced spacing, the nucleosome may or may not support TFIIIC binding and interaction with the TFIIIB upstream. Our earlier measurements on a template with multiple operator sites for the binding of a lac repressor found that for a nucleosome to translationally and symmetrically position between two protein-binding sites, a minimum of 165 bp should be freely available such that a 145-bp core DNA length leaves 10 bp free DNA room from the protein binding sites at both ends (Pusarla et al., 2007). Therefore, we predict that the nucleosome position between A and B boxes may remain unaltered till 35 bp deletions, while on d40, d45, and d50 it may be difficult to fit in, which may hamper the juxtaposing of the boxes. This nay result in inefficient TFIIIC binding and loss of transcription, as observed in this study.

Further deletions may either include the A/B boxes in the core DNA wound over the nucleosome making TFIIIC/B binding non-productive, or the TFIIIC binding may exclude the nucleosome, alleviating the chromatin repression. The increase in TFIIIC-dependent ND transcription on d55, d60, and d65 plasmids may be explained by the absence of the interfering pseudoA boxes, whereas the opposite results on the chromatin may be the outcome of two effects. First, the TFIIIC binding may lead to nucleosome exclusion but a steric obstruction of the gene body may reduce transcription. Alternatively, in the TFIIIC absence, the nucleosome may be found only downstream of the +85 bp position, as seen earlier *in vivo* (Marsolier et al., 1995). This would enable the gene to be transcribed as naked DNA, without chromatin repression.

### Nhp6 and T7 Effects on U6 Transcription are Manifested via TFIIIC and TFIIIB

The chromatin footprint on *SNR6* in a strain with deletion of 42 bp between boxes A and B, was found similar to that in a strain with a lethal point mutation on box B (Gerlach et al., 1995). The recognition of box A by TFIIIC in *SNR6* is reported to be an inefficient step during transcription complex assembly *in vitro* (Gerlach et al., 1995) and Nhp6A is shown to stabilize the TFIIIC-box A interaction (Kassavetis and Steiner 2006). A positive effect of Nhp6 specifically on *SNR6* transcription and synthetic lethality of Nhp6 with a 42 bp deletion between the terminator and box B, reducing the distance between boxes A and B to the near subnucleosomal size (Kruppa et al., 2001; Lopez et al., 2001; Martin et al., 2001), synthetic lethality with *SNR6* TATA box mutations (Gerlach et al., 1995), and *nhp6ΔΔ* condition (Martin et al., 2001), all could be explained by increased TFIIIC binding and single-round transcription with Nhp6 addition to *SNR6* *in vitro* (Kruppa et al., 2001). The *in vivo* chromatin structure altered around the TATA box region of *SNR6* in the nhp6ΔΔ cells was taken as an indication of altered TFIIIB binding, which could be a reason for the transcriptional repression of *SNR6* (Lopez et al., 2001). Therefore, the reduced transcription in deletion clones could be due to a loss or non-productive TFIIIC/TFIIIB binding to *SNR6*. This may be the reason that earlier a deletion of 42 bp between the terminator and box B showed synthetic lethality with several other promoter mutations in *SNR6* (Gerlach et al., 1995).

The T_7_ mutations do not abolish the TFIIIB footprint but show lethality in the absence of Nhp6 (Martin et al., 2001). While the TATA box and T_7_ stretch are found near the exit point of DNA in the US nucleosome, box A sits close to the DNA entry spot in the A–B box nucleosome. Nhp6 is generally found in the NFR near the entry/exit points of nucleosomal DNA (Dowell et al., 2010) and Nhp6A/B can cause looping and bending of DNA by at least 90° (Paull and Johnson 1995). Together, these observations raise the possibility that Nhp6 might be recruited to the T_7_ stretch, just upstream of TSS and stabilize the TFIIIC interaction with box A in turn. This is consistent with the highest association of Nhp6 with TFIIIC, out of all the components of the pol III transcription complex (Bhalla et al., 2019; Shukla et al., 2021). The inherent rigidity of a stretch of T’s confers inflexibility to DNA, which may allow their presence only at the entry/exit or dyad axis positions in the nucleosome. Thus, T_7_ may interfere with the encroachment of NFR by the US regulatory nucleosome on *SNR6* (Arimbasseri and Bhargava 2008).

### Nhp6 Influence on *SNR6* Transcription *in vivo* is Repressive

Transcription was found refractory to ∼300 ng Nhp6, whereas after saturation at ∼100 ng, higher Nhp6 additions inhibited chromatin transcription (Mahapatra et al., 2011). No transcription inhibition of *SNR6* ND was seen even up to 500-ng Nhp6 addition (Kruppa et al., 2001), whereas we found more than 180 ng Nhp6 as inhibitory for chromatin transcription *in vitro*. At lower levels, it caused even activation by enhancing the +1 transcription initiation. The requirement of both the upstream T_7_ stretch and TFIIIC for transcription activation by Nhp6 implies a balancing role for the T_7_ element in the dose-dependent effects of Nhp6. As Nhp6A is an abundant, non-sequence-specific DNA-binding protein, its effects may easily be dose-dependent *in vivo*. Increased Nhp6A occupancy on the *SNR6* gene under repression is consistent with a role for Nhp6 in further establishing the repressed chromatin state of *SNR6*.

Yeast *SNR6* is regulated by its unique chromatin organization and targeted by a plethora of epigenetic regulatory complexes (Bhargava 2013). This study shows that TFIIIC sequestration by pseudoA boxes, difficulty in chromatin formation, or TFIIIC binding due to distance/phase differences between A and B boxes also influence the *SNR6* transcription. The effects are individually small but subtle and significant when together. Enhancing transcription activation on the *SNR6* chromatin by Nhp6 is the outcome of a combined influence of TFIIIC and the T_7_ element on chromatin transcription while T_7_ stretch also affects TFIIIC binding. It appears that every part of the *SNR6* gene sequence has evolved with a unique role in fine-tuning its chromatin expression levels, making *SNR6* a specific target for Nhp6.

## Data Availability

The original contributions presented in the study are included in the article and Supplementary Files, further inquiries can be directed to the corresponding author.
