# Supplementary material for: Structural Features of the Nucleosomal DNA Modulate the Functional Binding of a Transcription Factor and Productive Transcription
Source: Front Genet. 2022 May 13;13:870700. doi: 10.3389/fgene.2022.870700 (PMC9136082; doi:10.3389/fgene.2022.870700)
Supplement: Supplementary file 1 [file DataSheet1.pdf]

Supplementary Material

for the manuscript

Structural features of the nucleosomal DNA modulate the functional binding of a transcription factor and productive transcription

Vinesh Vinayachandran and Purnima Bhargava*

List of the Contents of this File

I. Supplementary Tables S1 and S2

II. Supplementary Figure Legends

III. Supplementary Figures S1, S2, S3

I Supplementary Tables

Table S1 List of yeast strains used in this study

Strain	Phenotype	Genotype	Source
MHY308	H4 gene under its own promoter	*MATa, ade2-101, his3-Δ200, leu2-3,-112, lys2-801, trp1-Δ901, ura3-52, GAL^{+}, thr^{-}, tyr^{-}, arg4-1, Δh4-1, [HIS3^{+}], Δh4-2 [LEU2^{+}]/pUK499(TRP1^{+}, H4-2^{+})*	Han and Grunstein (1988)
UKY403	H4 gene under GAL1 promoter	*MATa, ade2-101, his3-Δ200, leu2-3,-112, lys2-801, trp1-Δ901, ura3-52, GAL^{+}, thr^{-}, tyr^{-}, arg4-1, Δh4-1, [HIS3^{+}], Δh4-2 [LEU2^{+}]/pUK421(TRP1^{+}, GAL1-H4-2^{+})*	Kim et al. (1988)
YPB14	Nhp6A-HA	*MATa ade2-1 his3-11,15 leu2-3,112 trp1-1 ura3-1 can1-100* Nhp6A-6XHA:KanMX4	This study
YPB15	Nhp6B-HA	*MATa ade2-1 his3-11,15 leu2-3,112 trp1-1 ura3-1 can1-100* Nhp6B-6XHA:KanMX5	This study

Han, M. and Grunstein, M. (1988) Nucleosme loss activates yeast downstream promoters in vivo. Cell 55, 1137-1145. doi: 10.1016/0092-8674(88)90258-9.

Table S2

Changes made between the Terminator and Box B in order to reduce the distance between the Boxes A and B in 5 bp increments

Sl. No	Plasmid Name	Deletion position in the gene; total bp deleted	Effective deletion/ Replaced with	Distance between; outcome		Comments
				Termin-BoxB	A-B boxes	
1.	pCS6	N.A.	0bp/ None	114 bp	202	
2.	pD5	+172 to +178; 7 bp	5 bp/ TG	109	197	Downstream of pseudoA boxes
3.	pD10	+170 to +182; 12 bp	10 bp/ AG	104	192	Downstream of pseudoA boxes
4.	pD15	+169 to +186; 18 bp	15 bp/ GGA	99	187	Downstream of pseudoA boxes
5.	pD20	+165 to +186; 22 bp	20 bp/ TG	94	182	Downstream of pseudoA boxes
6.	pD25	+165 to +190; 26 bp	25 bp/ T	89	177	Downstream of pseudoA boxes
7.	pD30	+156 to +190; 34 bp	30bp/ ACGC	84	172	4bp deletion in 2nd pseudoA box
8.	pD35	+157 to +194; 38 bp	35bp/ ATC	79	167	4bp deletion in 2nd pseudoA box
9.	pD40	+158 to +198; 41 bp	40 bp/ C	74;	162	3bp deletion in 2nd pseudoA box
10.	pD45	+149 to +108; 50 bp	45 bp/ AGATC	69	157	5bp deletion in 1st and complete deletion of 2nd pseudoA box
11.	pD50	+143 to +198; 56 bp	50 bp/ GATATC	64	152	Both pseudoA boxes deleted
12.	pD55	+143 to +199; 57 bp	55 bp/ CG	59	147	Both pseudoA boxes deleted
13.	pD60	+143 to +202; 60 bp	60 bp/ None	54	142	Both pseudoA boxes deleted
14.	pD65	+143 to +207; 65 bp	65 bp/ None	49	137	Both pseudoA boxes deleted
15.	pD70	+140 to +209; 70 bp	70 bp/ None	44	132	Both pseudoA boxes deleted
16.	pDT7	Only the T7 stretch replaced with ACGCACG. NO other change				T7 sequence disrupted

Deletions were made in the plasmid pCS6 harboring the yeast *SNR6* gene DNA sequence -120 to +312 with respect to transcription initiation site, from the genomic locus (Brow and Guthrie 1990). The U6 snRNA coding sequence was not changed in any of them. The sequence changes were made via the primer used for PCR amplification of the modified sequence using pCS6 as the template. A few bp changes were made as and when required for making the precise bp deletions between the terminator and box B. The 5 bp incremental deletion was made in the pCS6 DNA at the given position by replacing the pCS6 sequence with the modified sequence. The deletions could be classified into two groups; as **shorter deletions** (d5 to d40) and **longer deletions** (d45 to d65).

II Supplementary Figure Legends

Figure S1 Deletion of the DNA between the terminator and Box B shortens the distance between the boxes A and B as well as deletes eventually the pseudoA boxes. **(A)** Schematic representation of the *SNR6* gene organization in d45 (pD45) and d50 (pD50) as compared to that in pCS6. Depictions of different elements are explained by the legends given at the bottom of the panel. Numbers mark the positions of the elements in bp. **(B)** Disruption of the T_7 sequence in pCS6 by replacing it with a random sequence (both in red) to give the plasmid pU6dT_7 (dT_7). TATA box sequence and the +1 nucleotide are put in bold letters. **(C)** Comparison of naked DNA transcriptions of all deletion clones in the presence/absence of TFIIIC, all normalized against the respective pCS6 levels. Periodicity according to 5bp deletion is visible. **(D)** Gel shows comparison of naked DNA transcription from the plasmids pCS6, d50 and d55 in the absence and presence of TFIIIC. Duplicate samples are shown. Arrowhead marks the position of the correctly initiated transcript. **(E)** Quantifications of the chromatin from all the clones, normalized to pCS6 levels in the presence/absence of TFIIIC. Average and scatter of relative transcription levels from three independent experiments are shown. Asterisks mark only those showing a significant differences between the TFIIIC- and TFIIIC+ transcription. The p value for d20=0.014, d55=0.023 and d65=0.004. **(F)** A representative gel image showing the chromatin transcription of deletions upto 45 bp, with/without TFIIIC additions. The downstream initiations on all of them (marked by arrow heads) indicate perturbation in the right placement of the PIC

Figure S2 Nhp6 influences transcription of deletion clones differently.
Measured transcript levels were obtained by first normalizing with the recovery marker (R.M.) and then with corresponding pCS6 levels. **(A)** TFIIIC-dependent chromatin transcription of d40 and d45 is affected by Nhp6 in dose-dependent manner. A limited range addition of Nhp6 (60, 120, 180 ng) was used to titrate the chromatin templates in the presence of TFIIIC. Transcript and recovery marker positions are marked. Fold activation over no Nhp6 control in each lane is given at the bottom of the panel. On both the templates, the comparatively very low TFIIIC-dependent transcription in the absence of Nhp6 is highly increased with addition of even 60 ng Nhp6. The level of activation decreases gradually as added Nhp6 is increased. The effect is also unequal on both the templates. **(B)** Effect of 180 ng Nhp6 addition on transcription of naked and chromatinized pCS6, d5–d20 plasmids *in vitro*. Ratio of transcript levels in the presence and absence of Nhp6 (Nhp6 +/-) was taken as read-out of Nhp6 effect on either TFIIIC+ or TFIIIC- condition. Values of Nhp6 +/- ratios below 1 denote repression by Nhp6 whereas those above denote activation over the transcription level in the absence of Nhp6. Nhp6 supports the TFIIIC-dependent transcription of pCS6, d5, d20 but not of d15 and d10. **(C)** Quantification results of Nhp6 effect on ND transcription of pCS6 and d40-d65 plasmids. Ratios of transcription with (180 ng) and without Nhp6 in the absence or presence of TFIIIC are plotted. High scatter in most of the cases is due to very low (close to background) level of transcription, which makes measurements difficult. **(D)** ND transcription for pCS6, d40 and d45 with (120 ng) and without Nhp6 in the absence or presence of TFIIIC are shown. **(E)** Downstream initiations of chromatin transcription on deletion clones d50, d55 are not corrected by 120 ng Nhp6. Rightly initiated transcript is marked by +1 whereas downstream initiations from +5 and +7 bp positions are represented by the bands below the +1 transcript.

Figure S3 Disruption of T_7 promoter element increases the *SNR6* transcription
Nhp6 dose-dependence of chromatin transcription. Transcription of pCS6 and dT_7 chromatin in the absence or presence of TFIIIC and added Nhp6 are shown in this representative gel.

Samples loaded in the lanes 3 and 14 were swapped by mistake and correctly labeled here. Measured transcript levels in each lane are given at the bottom of the panel.

III. Supplementary Figures S1, S2, S3

Figure S1

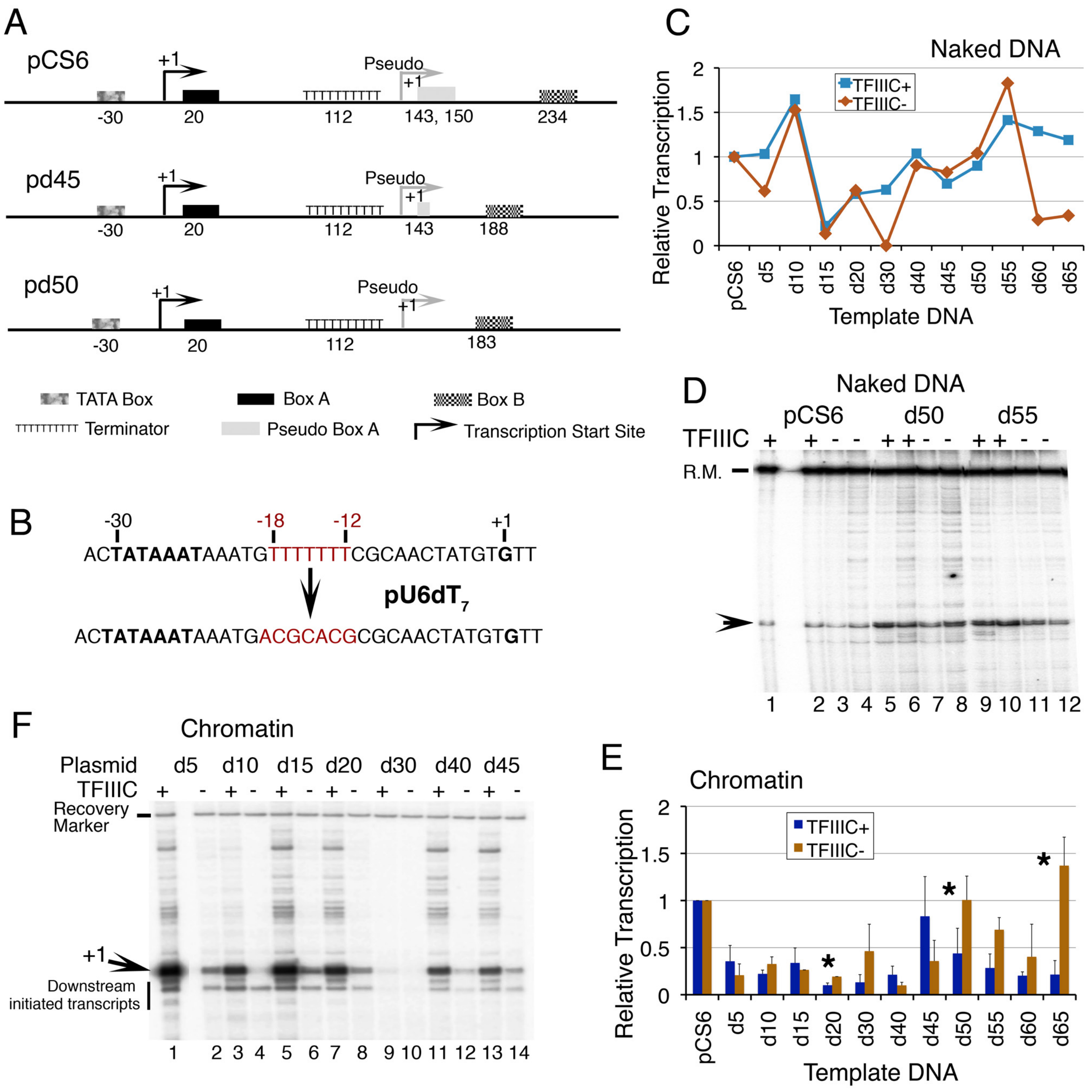

A
pCS6
+1
Pseudo
+1
-30
20
112
143, 150
234
pd45
+1
Pseudo
+1
-30
20
112
143
188
pd50
+1
Pseudo
+1
-30
20
112
183
TATA Box
Box A
Box B
Terminator
Pseudo Box A
Transcription Start Site
B
-30
-18
-12
+1
ACTATAAATAAATGTTTTTTTCGCAACTATGTGTT
pU6dT7
ACTATAAATAAATGACGCACGCGCAACTATGTGTT
C
Naked DNA
TFIIIC+
TFIIIC-
Relative Transcription
2
1.5
1
0.5
0
pCS6
d5
d10
d15
d20
d30
d40
d45
d50
d55
d60
d65
Template DNA
D
Naked DNA
pCS6
d50
d55
TFIIIC
+ + - - + + - - + + - -
R.M.
1 2 3 4 5 6 7 8 9 10 11 12
F
Chromatin
Plasmid
d5
d10
d15
d20
d30
d40
d45
TFIIIC
+ - + - + - + - + - + - + -
Recovery
Marker
+1
Downstream
initiated transcripts
1 2 3 4 5 6 7 8 9 10 11 12 13 14
E
Chromatin
TFIIIC+
TFIIIC-
Relative Transcription
2
1.5
1
0.5
0
pCS6
d5
d10
d15
d20
d30
d40
d45
d50
d55
d60
d65
Template DNA

Figure S2

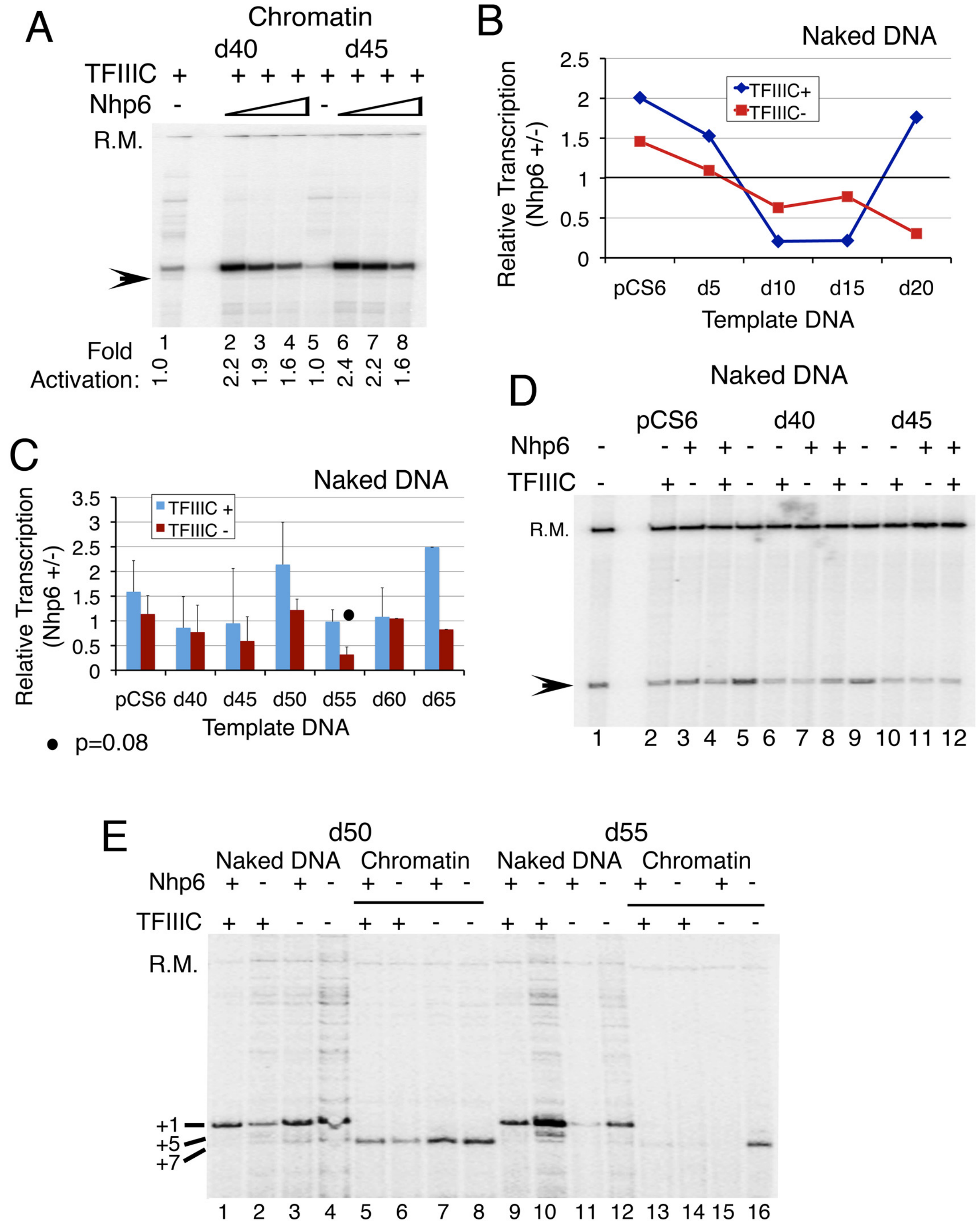

A
Chromatin
d40
d45
TFIIIC
Nhp6
R.M.
Fold Activation:
1 2 3 4 5 6 7 8
1.0 2.2 1.9 1.6 1.0 2.4 2.2 1.6
B
Naked DNA
Relative Transcription (Nhp6 +/-)
TFIIIC+
TFIIIC-
pCS6 d5 d10 d15 d20
Template DNA
C
Naked DNA
Relative Transcription (Nhp6 +/-)
TFIIIC +
TFIIIC -
pCS6 d40 d45 d50 d55 d60 d65
Template DNA
● p=0.08
D
Naked DNA
pCS6
d40
d45
Nhp6
TFIIIC
R.M.
1 2 3 4 5 6 7 8 9 10 11 12
E
d50
d55
Naked DNA
Chromatin
Naked DNA
Chromatin
Nhp6
TFIIIC
R.M.
+1
+5
+7
1 2 3 4 5 6 7 8 9 10 11 12 13 14 15 16

Figure S3

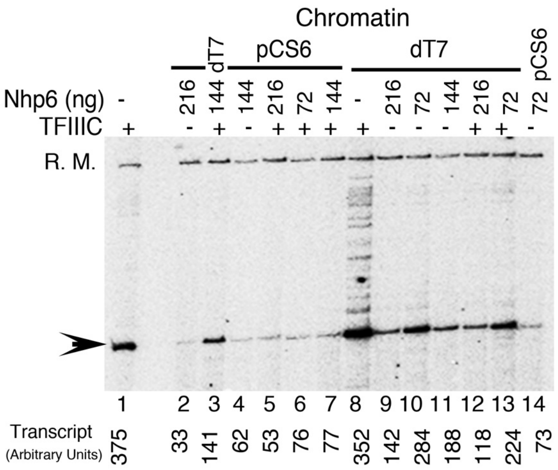
Chromatin
dT7
pCS6
dT7
pCS6
Nhp6 (ng)
TFIIIC
R. M.
1 2 3 4 5 6 7 8 9 10 11 12 13 14
Transcript
(Arbitrary Units)
375 33 141 62 53 76 77 352 142 284 188 118 224 73
